# Hypoxia regulates GR function through multiple mechanisms involving microRNAs 103 and 107

**DOI:** 10.1016/j.mce.2020.111007

**Published:** 2020-12-01

**Authors:** Nan Yang, Andrew Berry, Carolin Sauer, Matthew Baxter, Ian J. Donaldson, Karen Forbes, Rachelle Donn, Laura Matthews, David Ray

**Affiliations:** a Faculty of Biology, Medicine, and Health, University of Manchester and Manchester Academic Health Sciences Centre, Manchester, M13 9PT, UK; b NIHR Oxford Biomedical Research Centre, John Radcliffe Hospital, Oxford, OX3 9DU, UK; c Oxford Centre for Diabetes, Endocrinology and Metabolism, University of Oxford, Oxford, OX37LE, UK

**Keywords:** Glucocorticoids, Glucocorticoid receptor, MicroRNAs, Hypoxia, HIF-1α

## Abstract

Glucocorticoids (Gcs) potently inhibit inflammation, and regulate liver energy metabolism, often acting in a hypoxic environment. We now show hypoxic conditions open a specific GR cistrome, and prevent access of GR to part of the normoxic GR cistrome. Motif analysis identified enrichment of KLF4 binding sites beneath those peaks of GR binding exclusive to normoxia, implicating KLF4 as a pioneer, or co-factor under these conditions. Hypoxia reduced KLF4 expression, however, knockdown of KLF4 did not impair GR recruitment. KLF4 is a known target of microRNAs 103 and 107, both of which are induced by hypoxia. Expression of mimics to either microRNA103, or microRNA107 inhibited GR transactivation of normoxic target genes, thereby replicating the hypoxic effect. Therefore, studies in hypoxia reveal that microRNAs 103 and 107 are potent regulators of GR function. We have now identified a new pathway linking hypoxia through microRNAs 103 and 107 to regulation of GR function.

## Highlights

• Oxygen tension profoundly affects glucocorticoid (Gc) action.
• Gc target genes and regulatory elements are sensitive to oxygen tension.
• MicroRNAs 103 and 107 mediate the hypoxic regulation of Gc action.
• Hypoxia does not affect Gc receptor (GR) expression, modification or trafficking.

## 1 Introduction

Hypoxia is a condition observed in many diseases, such as inflammation. The tissue response to hypoxia is dominated by the actions of the hypoxia-inducible factors (Moslehi and Rathmell, 2020). These transcription factors are stabilized at the protein level under conditions of low oxygen tension, and they direct an integrated response, with many down-stream genes showing changes in expression, and also impacts on the expression of non-coding RNAs, such as the microRNAs 103 and 107 (Kulshreshtha et al., 2007). Specifically, the HIF-1α transcriptional subunit is recognized by prolyl hydroxylases and targeted for degradation via the Von Hippel Lindau (VHL)-mediated ubiquitin proteasome pathway. Under hypoxic conditions HIF-1α is stabilized and translocates to the nucleus to exert its transcriptional activity.

As the most potent anti-inflammatory agents, glucocorticoids (Gcs) are regularly used in clinic to treat a range of inflammatory diseases, such as asthma and rheumatoid arthritis. However, there remain major issues in the widespread use of therapeutic glucocorticoids resulting from insensitivity and off-target effects. It would be very useful to understand why inflammatory diseases frequently require high-dose, high potency glucocorticoid treatment to respond (Yang et al., 2012). One possible factor at sites of active inflammation is reduced oxygen tension.

Gcs act through the ubiquitously expressed glucocorticoid receptor (GR), a ligand activated transcription factor and member of the nuclear receptor superfamily. GR function is affected by changes to the local tissue microenvironment (Yang et al., 2012). Therapeutically, there is marked variation in response to standard doses of Gc, which has been attributed to the expression of cytokines at the sites of inflammation (Ishiguro, 1999; Leung et al., 1995; Matthews et al., 2004), and also to the local hypoxic environment, caused by tissue pressure from edema, disruption of local blood supply, and increased oxygen consumption by infiltrating cells. Hypoxia affects local expression of cytokines, including IL-8 and macrophage migration inhibitory factor, but may also directly influence GR function.

To date, studies defining the impact of cell hypoxia on GR function have been inconclusive. Hypoxic preconditioning is reported to induce GR expression and increase glucocorticoid sensitivity (Kodama et al., 2003; Leonard et al., 2005; Sengupta and Wasylyk, 2004; Wang et al., 2012). However, hypoxia is also reported to reduce GR-dependent gene expression (Charron et al., 2009; Wagner et al., 2008). These contrasting findings indicate the presence of highly dynamic interactions between GR function and oxygen tension, possibly mediated through HIF-1α. Such interactions may influence adaptation to hypoxic environments. Recent advances in genomic technology provide a broader opportunity to study multiple levels of control of gene expression. GR occupies only a small subset of its potential binding sites across the genome in any single cell-type, resulting in strong cell-type specific control over GR function. Indeed, the overlap of Gc regulated gene expression profiles between observed cell lines is modest (John et al., 2009; Rogatsky et al., 2003; Wang et al., 2004). Epigenetic regulation mechanisms may be key to this, including nucleosome positioning, histone modification, and possibly DNA modification (Wiench et al., 2011a).

More recent work has suggested that in turn glucocorticoid action may impact on HIF function. It appears that activated GR affects the stability of the VHL protein, and that as a result HIF-1α is stabilized, even under normoxic conditions. This suggests that some of the actions of glucocorticoids may in part be due to activation of HIF-1α action (Vettori et al., 2017).

Here, we use ChIP-seq to map the GR cistrome under normoxic and hypoxic conditions. Our findings reveal that enrichment of KLF4 motif under GR peaks in normoxia is lost in hypoxia, but KLF4 knockdown alone did not affect GR function. KLF4 is targeted by the hypoxia-induced microRNAs 103 and 107; both of which were indeed induced by hypoxia in our system. Mimetics to these microRNAs attenuated GR action, when used alone or in combination. Therefore microRNAs 103 and 107 emerge as potent regulators of the GR in hypoxia.

## 2 Materials and methods

### 2.1 Antibodies and plasmids

Anti-GR (clone 41) was from BD Biosciences (Oxford, UK); anti-GR (M-20 and H-300) and rabbit IgG were from Santa Cruz Biotechnology, anti-GR (HPA004248), anti-α-tubulin, hypoxia-mimetic deferoxamine and dexamethasone were from Sigma-Aldrich (Dorset, UK); anti-GR (24050-AP-1) was from Proteintech (Manchester, UK); anti-phospho-(Ser211)-GR was from Cell Signalling Technology (MA, USA); anti-H3K27ac was from Millipore; horseradish peroxidase conjugated anti-mouse and anti-rabbit were from GE Healthcare (Buckinghamshire, UK); fluorophore conjugated (Alexaflour 488) anti-mouse was from Invitrogen molecular probes (Paisley, UK); TAT3-luc a kind gift of Dr J Lluihi-Ineguez, University of California, San Franscisco, CA, USA. The NRE–luciferase reporter plasmid was obtained commercially (Stratagene). hGR pcDNA3 are a kind gift of Dr. M Norman, University of Bristol, Bristol, UK. GR deletants N500 and AF1 have been described previously (Berry et al., 2010). The HIF-1α expression vector was a kind gift from Dr. Costas Demonacos.

### 2.2 Cell culture and maintenance

Human cervical carcinoma cells and human embryonic kidney cells (HeLa and HEK; ECACC, Wiltshire, UK) were maintained at 37 °C with 5% CO_2_ in a humidified atmosphere and cultured in DMEM medium supplemented with GlutaMAX and 10% FCS (Invitrogen, Paisley, UK). In order to ensure no residual Gcs were present during experiments, cells were grown in media supplemented with 10% charcoal dextran stripped serum (Invitrogen) for 24 h prior to treatment. For experiments conducted under anoxic culture conditions, HeLa and HEK cells were transferred to an anaerobic chamber (Bactron anaerobic chamber, Sheldon Manufacturing, Cornelius, OR, USA) supplied with a gas mixture of 5% CO_2_:5% H_2_:90% N2, which removes residual oxygen by passing over a palladium catalyst. Exposures to hypoxia were generated using continuous gassing of a sealed chamber containing 1% oxygen with 5% CO2 in nitrogen. Oxygenation was assessed by an oxygen meter (measurement accuracy, ±1%; WPI Inc. Sarasota, FL).

### 2.3 Reporter gene assay

Cells were transfected with 2 μg of TAT3-luciferase and 0.5 μg of CMV-Renilla luciferase reporter using FuGENE 6 (3:1 Fugene: DNA ratio, Roche Diagnostics, Indianapolis, IN, USA). After 24 h, cells were transferred to medium containing charcoal dextran-stripped serum, treated before lysis, and then assayed for luciferase activity following the manufacturer's instructions (Promega). To control for transfection efficiency, cells were taken from a single pool and divided into different treatment conditions. All firefly luciferase readings were normalised to *Renilla* luciferase.

### 2.4 Quantitative RT-PCR

After dexamethasone (Sigma-Aldrich, D4902) treatment, total RNA was prepared from HeLa cells using RNeasy Mini Kit with on-column DNase I digestion (Qiagen, Valencia, CA, USA) and cDNA was synthesised using High Capacity RNA to cDNA kit and analyzed using Power SYBR Green PCR Master Mix (Applied Biosystems, CA, USA). GAPDH was used as an endogenous control. The expression of KLF4 level was detected using TaqMan gene expression assay (Applied Biosystems, Foster City, CA, USA). 18S rRNA was used as an endogenous control for multiplex reactions. Expression levels were calculated using the comparative C_t_ method, normalising to the control.

### 2.5 Immunoblot analysis

Cells were lysed in NETN buffer (0.5% NP-40, 1 mM EDTA, 50 mM Tris-Cl (pH 8.0), NaCl (120 mM) containing protease (Calbiochem, San Diego, CA, USA) and phosphatase inhibitors (Sigma-Aldrich). Proteins were separated by SDS gel electrophoresis and transferred to 0.2 μM nitrocellulose membranes (Bio-Rad Laboratories, Hertfordshire, UK) overnight at 4 °C. Membranes were blocked for 6 h (0.15M NaCl, 1% milk and 0.1% Tween 20) and incubated with primary antibodies (diluted in blocking buffer) overnight. Following three washes (88 mM Tris, pH 7.8, 0.25% dried milk, and 0.1% Tween 20), membranes were incubated with a species-specific horseradish peroxidase–conjugated secondary antibody (in wash buffer) for 1 h at room temperature and washed a further three times, each for 10 min. Immunoreactive proteins were visualised using enhanced chemiluminescence (ECL Advance, GE Healthcare). All primary immunoblot images are available in Fig. S5.

### 2.6 Immunofluorescence

Cells were washed twice in PBS then fixed with 4% paraformaldehyde (PFA) for 30 min, then permeabilised (0.02% Triton X-100 in PBS) for 30 min at room temperature (RT). Fixed cells were blocked (1% FCS, 0.01% Triton X-100 in PBS) and incubated with primary antibody overnight at 4 °C. After three washes with PBS cells were incubated in secondary antibody for 2 h. After incubation with Hoechst for 5 min, coverslips were washed three times with PBS and mounted using Vectashield aqueous HardSet mountant (Vector Laboratories, Peterborough, UK). Images were acquired on a Delta Vision RT (Applied Precision) restoration microscope using a 60x/1.42 Plan Apo objective and the Sedat filter set (Chroma 89000) and collected using a Coolsnap HQ (Photometrics) camera with a Z optical spacing of 0.5 μm. Raw images were deconvolved using the Softworx software and maximum intensity projections of deconvolved images processed using ImageJ.

### 2.7 Co-immunoprecipitation

HeLa cells transfected with HIF-1α, treated with vehicle or dexamethasone for 1 h then lysed in RIPA buffer containing protease and phosphatase inhibitors. GR was immunoprecipitated using GR-antibody (or IgG as a control) complexed to Protein A Sepharose beads overnight at 4 °C. Beads were collected by centrifugation (20 s at 2000g), washed three times with PBS and then boiled in SDS loading buffer prior to electrophoresis.

### 2.8 Chromatin immunoprecipitation (ChIP)

ChIP assays were performed as per standard protocols (Elsby et al., 2009). Cells were treated with either 100 nM dexamethasone or DMSO as control for 1 h, and then cross-linked with 1% formaldehyde at room temperature for 10 min. The reaction was quenched by adding 0.125 M glycine for 5 min. Following collection by low-speed centrifugation (700 g), nuclei were lysed (50 mM Tris-HCl pH 8.1, 10 mM EDTA, 1% SDS and protease inhibitor) and sheared twice by probe sonication (SONICS VCX130) with energy more than 50% (12 s on, 40sec off), followed by water sonication using Bioruptor (Diagenode) for 40 min, at 4 °C (high level, 30 sec on, 30 sec off).

The size of sheared DNA fragments were between 100 bp and 500 bp, with most approximately 200 bp. Immunoprecipitated chromatin (IP) was 1 in 10 diluted using IP dilution buffer (16.7 mM Tris-HCl pH 8.1, 1.2 mM EDTA, 167 mM NaCl, 0.01% SDS, 1.1% Triton X-100 and protease inhibitor). Each IP was incubated in 3 μg GR antibody (1 μg HPA004248, 1 μg M-20, 1 μg H-300), or 3 μg YFP antibody as control at 4 °C overnight, and then Dynabeads Protein G (Invitrogen) added for 4 h with slow rotation. For the KLF4 knockdown ChIP experiment, precleared IP was added with either 2 μg GR antibody (24050-1-AP; Proteintech, Manchester, UK), or 20 ng of Drosophila Spike-In chromatin and 2 μg of corresponding Spike-In antibody (Active Motif, La Hulpe, Belgium) as control. Following overnight incubation, 10 μl of equilibrated MagReSyn® Protein G beads were added rotating at 20 rpm at 4 °C for 2 h.

Subsequently, the beads were washed twice with Buffer 1 (20 mM Tris-HCl pH 8.1, 2 mM EDTA, 50 mM NaCl, 0.1% SDS, 1% Triton X-100 and protease inhibitor), once with Buffer 2 (10 mM Tris-HCl pH 8.1, 1 mM EDTA, 250 mM LiCl, 1% NP40, 1% sodium deoxycholate and protease inhibitor) and twice with TE buffer (10 mM Tris-HCl pH 8.0, 1 mM EDTA pH 8.0 and protease inhibitor). Chromatin was eluted in 1% SDS and 100 mM NaHCO3, and then incubation in 200 mM NaCl at 65 °C overnight to reverse the crosslinks. The DNA was cleaned up using a MinElute PCR Purification Kit (28006; Qiagen, Valencia, CA, USA). Real-time quantitative PCR was performed using primers specific to TSC22D3, FKBP5, MT1X, IL6ST and BATF using SYBR Green detection system.

### 2.9 Sequencing and ChIP-seq analysis

GR ChIP-seq in HeLa cells were performed on an Illumina HiSeq 2000 genome analyzer by BGI, and the alignment of 49 bp sequences was based on the Human Reference Genome (assembly hg19, NCBI Build 37.1, February 2009) using SOAP version 2.21 (Li et al., 2009). Reads with SOAP alignment quality values less than 20 were removed. The unmapped and mapped reads are deposited with ArrayExpress (E-MTAB-1851). For each sample, 9.5 M equal best matching reads were used for peak calling; enriched regions were identified by comparing ChIP sample reads with input sample reads, using the MACS peak caller version 1.4.0 (Zhang et al., 2008). The region was defined as a peak where p-value < 1e-5.

### 2.10 Heatmap generation

In different samples GR binding regions were used as targets to generate the heatmaps of ChIP-seq data. The comparative analysis was completed using seqMINER_1.3.3e software (Ye et al., 2011). Read enrichment values within ± 5 kb of GR binding regions was assigned to each 25bp bin, which were linearly normalised to sequencing reads of control. KMeans was applied for clustering to four groups.

### 2.11 STRING 10 analysis

MicroRNA103–3p/107 targets were defined using Targetscan (Agarwal et al., 2015). The STRING database version 10.5 (Szklarczyk et al., 2017) was used to identify GR interactors within the network of Targetscan-defined list of microRNA targets. Interaction sources were limited to experimental evidence and databases. Line thickness indicates confidence of interaction.

### 2.12 Motif analysis

Motif discovery used RSAT (Thomas-Chollier et al., 2012). Sequences of 200bp centered on each GR summit were analyzed using peak motifs ChIP-seq analysis. Two of the available methods were applied, oligo-analysis and position-analysis, using the default background (Markov model adapted to sequence length). Discovered motifs were ranked by corrected p value or ‘Sig’ value. ‘Sig’ is defined as: ‘Sig’ = -log10 (e value), where e is the expected number of false positives corresponding to the motif p value. Pearson's correlation and motif width normalised correlation indicate the similarity of the discovered motifs to the transcription factor reference databases (Jaspar, RSAT).

### 2.13 Gene ontology (GO) analysis

GO function analysis of peak-related genes was performed using GREAT version 2.0.2 (McLean et al., 2010). Following the use of the default gene regulatory definition option GREAT identified binding regions up to 5 kb upstream, together with 1 kb downstream to each gene. The regulatory region was then extended in both directions up to 1000 kb. Core peaks were selected for analysis using false discovery rate (FDR) less than 0.1.

### 2.14 CEAS analysis

The genomic distribution the of binding sites was determined using CEAS (Shin et al., 2009). Promoter regions were defined as starting 3 kb upstream from RefSeq transcription start sites (TSS), binding regions > 3k bp away from TSS was considered to be distal intergenic.

### 2.15 Quantitative RT-PCR analysis of microRNAs

The miRcury LNA™ Universal RT microRNA PCR system (Exiqon) was applied to measure expression of microRNAs, using LNA enhanced specific primer sets for either hsa-miR-103a-3p (target sequence: AGCAGCAUUGUACAGGGCUAUGA), or hsa-miR-107 (target sequence: AGCAGCAUUGUACAGGGCUAUCA). Relative amount of individual microRNA was normalised to 5S rRNA. The fold change in microRNA expression was calculated using the comparative C_t_ method.

### 2.16 KLF4 knockdown via siRNA transfection

siRNA transfections were carried out using two pre-designed Silencer® Select siRNAs specific for KLF4 (s17794 and s17793, Ambion Life Technologies, Austin, TX, USA). Silencer® Select Negative Control No. 1 siRNA (Ambion-4390844) was used as a negative control. Lipid and siRNA complexes were prepared using DharmaFECT Transfection Reagent 1 (GE Dharmacon, Lafayette, CO, USA) in OptiMEM media (Gibco™ Thermo Fisher Scientific, Waltham, MA, USA) and diluted into 10 cm plates containing HeLa cells at a concentration of 5 × 10^5^ cells/plate 24 h prior to transfection. Final siRNA concentrations were set to 25 nM, and cells were transfected for a total of 72 h.

### 2.17 Statistical analysis

Data were analyzed using SPSS in multiple samples. Multiple means were compared by one-way ANOVA followed by Bonferroni *post hoc* test or Kruskal-Wallis followed by Dunn's *post hoc* test where Gaussian distribution could not be assumed. For comparison of two groups a Student's t-test for independent samples was used. For non-parametric data a Mann-Whitney *U* test was used. Statistically significant was considered as p-value < 0.05.

## 3 Results

### 3.1 Hypoxia rewires the GR cistrome

**Fig. 1:**
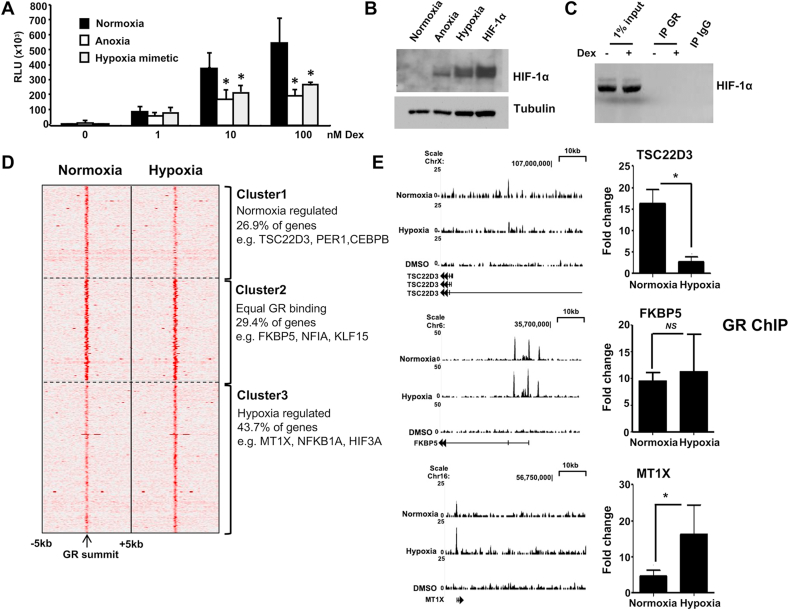
**Hypoxia rewires the GR cistrome**. (A) HeLa cells were transfected with 2 μg TAT3-luc, cultured under normoxic conditions in the presence or absence of the hypoxia mimetic deferoxamine (100 μM), or in an anoxic chamber then treated with dexamethasone (Dex) for 16 h prior to lysis and luciferase assay. (B) Cells cultured in normoxia, anoxia, treated with deferoxamine or transiently transfected with HIF-1α were lysed and immunoblotted for HIF-1α or tubulin as a loading control. (C) Cells treated with deferoxamine were incubated with or without 100 nM dexamethasone for 1 h, lysed, immunoprecipitated for GR, and then immunoblotted for HIF-1α. (D) HeLa cells were cultured in either normoxia or hypoxia overnight, and GR cistrome identified by ChIP-seq. The heatmap shows binding peak intensity of 595 core GR binding sites aligned according to their summits. Three clusters were applied, and 5 kb upstream and 5 kb downstream regions around the summit are plotted. (E) UCSC browser tracks for TSC22D3, FKBP5 and MT1X in normoxia and hypoxia, where the y axis of each track represents the coverage by non redundant and extended reads from MACS analysis. These RefSeq annotated genes are shown and the gene direction indicated by an arrowhead. GR binding sites were quantified by ChIP-qPCR. Graphs depict fold enrichment of immunoprecipitated GR in response to treatment with dexamethasone over vehicle treated control and show mean ± S.D. from three independent experiments. *p < 0.01, NS: not significant.

**Fig. S1:**
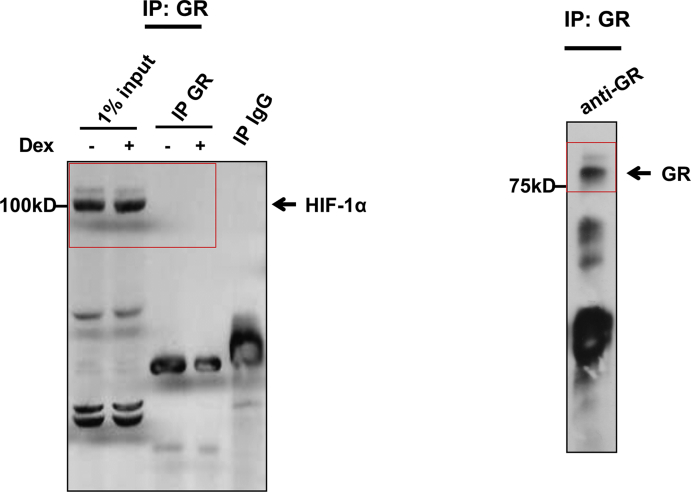
**Original scans of co-IP experiment in**Fig. 1**C.** HeLa cells treated with deferoxamine were incubated with or without 100 nM dexamethasone for 1 h, lysed, immunoprecipitated for GR, and then immunoblotted for either HIF-1α, or GR as a positive control.

Culture in hypoxia or treatment with a hypoxia-mimetic deferoxamine induced Gc resistance to a simple, transfected reporter gene (Fig. 1A). We confirmed the conditions induced stabilization of HIF-1α protein (Fig. 1B), but were unable to find evidence for a direct interaction between GR and HIF-1α by co-immunoprecipitation (Fig. 1C, Fig. S1).

To identify genome-wide binding of GR in response to dexamethasone in normoxic or hypoxic culture conditions, we took an unbiased ChIP-seq approach. We identified 595 core GR binding sites with high confidence that were dependent on ligand activation (Fig. 1D). There were differences in GR recruitment dependent on oxygen tension, with some sites lost, and others gained in hypoxic conditions (Fig. 1D). Identified GR binding sites were validated by ChIP-PCR. In line with the ChIP-seq analysis, ChIP-PCR shows reduced GR binding at TSC22D3 (or GILZ), equal GR binding at FKBP5 and increased binding at MT1X promoters in response to hypoxia (Fig. 1E).

### 3.2 Hypoxia retains similar GR distribution with different gene function

**Fig. 2:**
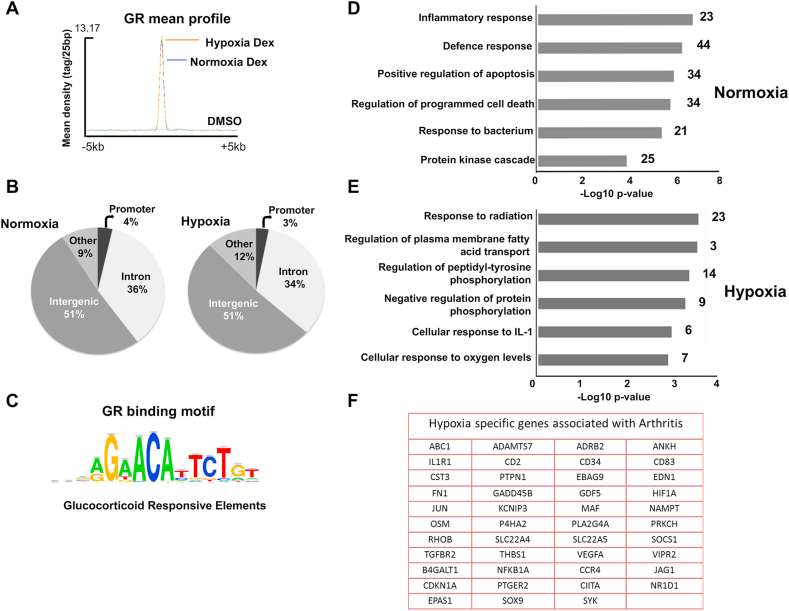
**Hypoxia retains similar GR distribution with different gene function**. (A) Average signal intensity of all core GR binding events for normoxia (blue) and hypoxia (orange). (B) Pie charts represent the genomic distribution of Gc induced GR-bound regions. (C) Motif discovery analysis identifies the enrichment of glucocorticoid responsive elements (GRE). (D, E) The analysis of GR binding regions and the corresponding gene ontology of their associated genes were carried out using GREAT. Significantly enriched terms from the biological process category are illustrated as bar charts for normoxia or hypoxia. (F) List of genes associated with arthritis identified as being regulated by GR binding events specifically in hypoxia.

Hypoxia did not alter the average signal intensity of GR binding in response to dexamethasone, again implying no major effect on GR expression or global alteration in GR function (Fig. 2A). The core 200bp regions centered on GR peak summits were subsequently analyzed using CEAS. We found more than 50% of the GR binding events located in distal intergenic regions (Fig. 2B, Table S1), in agreement with previous observations (Uhlenhaut et al., 2013). There was no significant difference in the location of GR binding sites in relation to coding regions between hypoxia and normoxia, and as expected the peaks of GR binding regions revealed enrichment of the consensus glucocorticoid responsive element (GRE) motif under both conditions (Fig. 2C).

We further analyzed likely genes regulated by the GR cistromes under normoxic and hypoxic conditions using GREAT (McLean et al., 2010). Hypoxia depleted GR target genes involved in gene ontology (GO) biological processes, including ‘inflammatory response’ (Fig. 2D, Table S2), and increased targets genes involved in the ‘response to oxygen’ (Fig. 2E). Disease ontology analysis identified several hypoxia-associated genes with significant terms involved in inflammatory diseases, e.g. arthritis (Fig. 2F, Table S3).

### 3.3 Hypoxia regulates H3K27 acetylation at GR binding sites

**Fig. 3:**
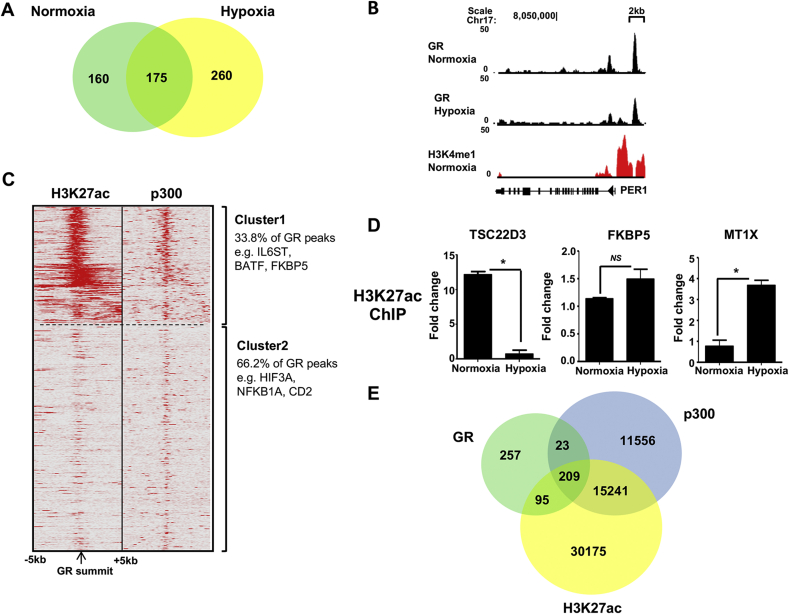
**Hypoxia regulates H3K27 acetylation at GR binding sites**. (A) A Venn diagram describes overlap of GR cistromes in normoxia (green) and hypoxia (yellow). (B) UCSC browser track for GR binding at the PER1 locus, where the y axis depicts the coverage by non redundant and extended reads from MACS analysis. (C) Cistromes for H3K27ac, and p300 in HeLa cells were aligned with GR binding peaks. Two groups were observed, one showing strong overlap amongst the three signals, and one cluster showing negligible overlap. (D) H3 acetylation levels of TSC22D3, FKBP5 and MT1X were quantified by ChIP-qPCR, in response to dexamethasone treatment. Graphs illustrate fold enrichment of immunoprecipitated H3K27ac in response to treatment with dexamethasone over vehicle treated control. Mean ± S.D. from three independent experiments. *p < 0.01, *NS*: not significant. (E) A Venn diagram summarises the overlap of the three cistromes, where GR is shown in green, p300 in blue and H3K27ac in yellow.

As oxygen tension specifically drives the distribution of GR binding sites, we compared the number of genes regulated in either normoxia or hypoxia, and found only 175 genes in both conditions (Fig. 3A). The majority of the sites identified in hypoxia were not seen in normoxia. This prompted more detailed examination at specific GR target sites, starting with the well-characterised PER1 locus. This analysis revealed a reduction in GR binding under hypoxic conditions (Fig. 3B), consistent with the observed loss of GR transactivation (Fig. 1A, D). To analyze the functional significance of the GR binding sites identified under normoxic conditions, we overlaid the GR cistrome with the histone H3K27ac and co-activator p300 ChIP-SEQ data, previously defined by the ENCODE project (GEO accessions GSM733684 and GSM935500). There was clear overlap between GR binding and p300, and also with enrichment of H3K27ac in 209 of the 595 high-confidence GR binding sites (Fig. 3C; Cluster 1). The p300 co-activator is a powerful mediator of GR transactivation, and brings histone acetylation enzymatic activity to the GR bound sites (Guo et al., 2017). In contrast, more than half of the GR binding sites showed negligible enrichment for the co-activator, and active histone mark (Fig. 3C; Cluster 2). This suggests identification of different classes of GR binding sites, potentially both enhancer and repressor elements (Fig. 3C).

To test if the differential acetylation of H3K27 was seen there, index target GR binding sites related to the GR-transactivated target genes TSC22D3, FKBP5 and MT1X were analyzed by ChIP-PCR (Fig. 3D). Indeed, these data showed that there was a loss of acetylated H3K27 in response to dexamethasone treatment in hypoxia for the TSC22D3 GR element, and in contrast a gain at the MT1X site. Hypoxia had no effect on H3K27 acetylation at the GR binding site on FKBP5. These changes in post GR activation chromatin state are predicted from the corresponding changes in GR recruitment to these sites (Fig. 1E), and reinforce the importance of H3K27ac as a mark of active chromatin at GR binding sites (Fig. 3C; Cluster 1). The different composition of DNA-bound complexes is illustrated by the analysis of co-binding between the GR and the co-modulator, and histone acetyltransferase p300, and that of its chromatin mark H3K27ac (Fig. 3E). The sites bound both by GR and p300 are far more likely to have an activating chromatin mark (H3K27ac) that those bound by GR alone, which may be a surrogate to identify genes repressed by GR.

### 3.4 Hypoxia eliminates the enrichment of KLF4 motif at GR binding sites

**Fig. 4:**
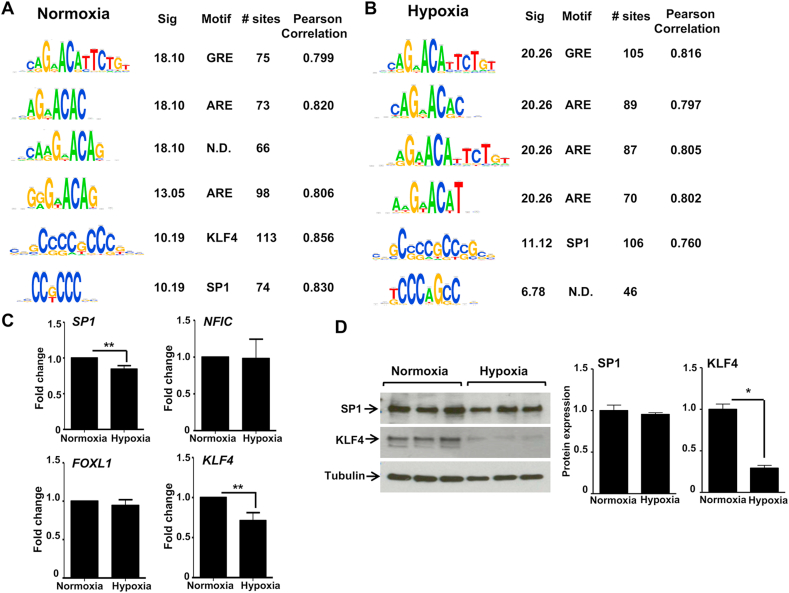
**Hypoxia eliminates the enrichment of KLF4 motif at GR binding sites**. (A, B) Motifs under peaks of GR binding in normoxia and hypoxia were analyzed using RSAT. The top 6 GR binding motifs identified were ranked by adjusted p value, ‘Sig’. Equally ranking motifs were ordered by decreasing percentage of GR binding regions containing the motifs. The degree of similarity between discovered motifs and matching motifs in JASPAR were measured by Pearson correlation coefficient. N.D. refers to the motifs were not discovered previously. (C) HeLa cells were cultured in either normoxia or hypoxia overnight. RNA samples were purified and the expression of SP1, NFIC, FOXL1 and KLF4 transcripts determined. (D) Protein samples were extracted from HeLa cells and KLF4 and SP1 expression measured by immunoblotting. Immunoblots show samples from three independent experiments. Immunoreactive bands for SP1 and KLF4 were quantified using Image J software, normalised to tubulin expression and then represented as a fold change over normoxia cultured cells. Graphs show mean ± S.D. of experiments repeated three times. **p < 0.01, *p < 0.05.

**Fig. S2:**
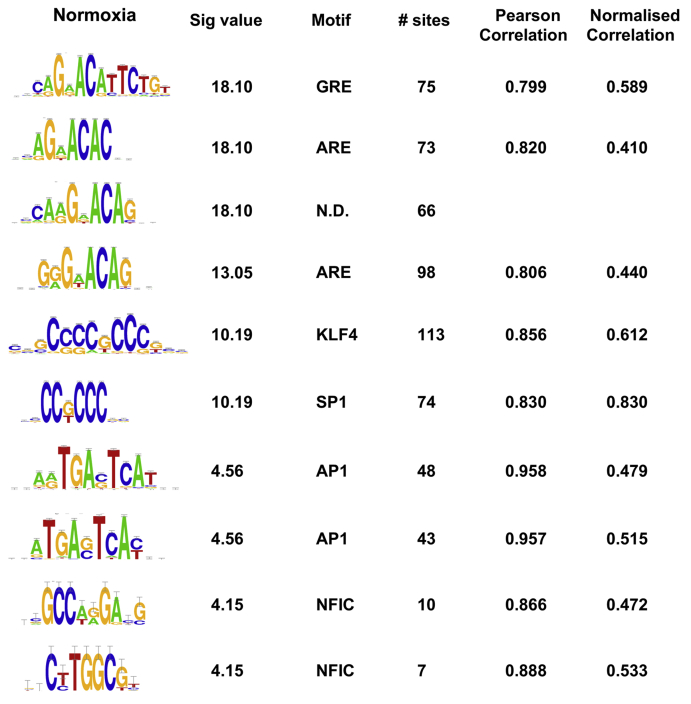
**Motif analysis of enriched GR regions in normoxia.** Top 10 motifs identified in normoxia were ranked by adjusted p value, ‘Sig’. Equally ranking motifs were ordered by decreasing percentage of GR binding regions containing the motifs. The degree of similarity between discovered motifs and matching motifs in JASPAR were measured by Pearson correlation coefficient and motif length normalised coefficient.

**Fig. S3:**
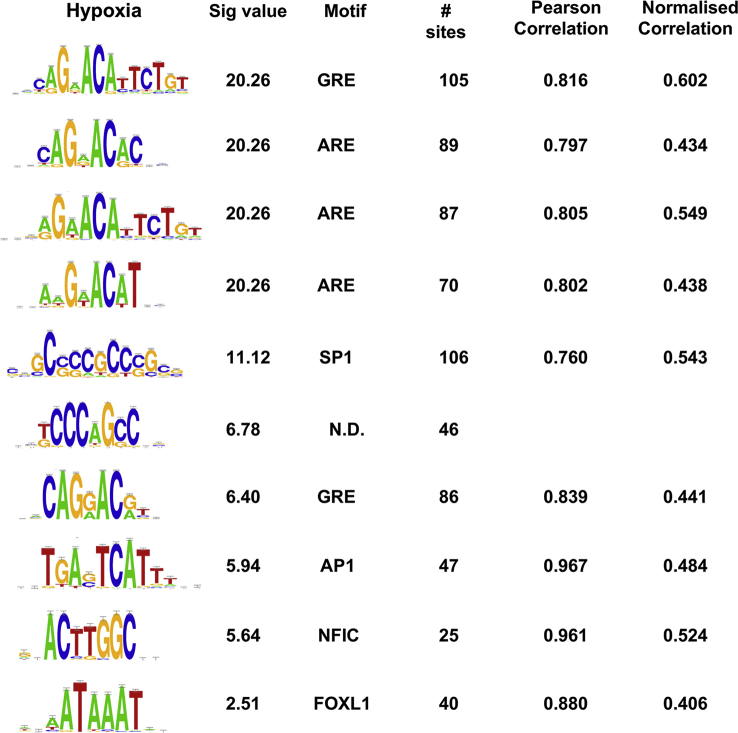
**Motif analysis of enriched GR regions in hypoxia.** Top 10 motifs identified in hypoxia were ranked by adjusted p value, ‘Sig’. Equally ranking motifs were ordered by decreasing percentage of GR binding regions containing the motifs. The degree of similarity between discovered motifs and matching motifs in JASPAR were measured by Pearson correlation coefficient and motif length normalised coefficient.

DNA motif analysis (RSAT) in the 200bp sequences centered on the summit of GR binding region identified GRE, ARE, SP1, AP1 and NF1C motifs in both normoxic and hypoxic groups (Fig. 4A and B, Figs. S2 and S3). We noted that potential KLF4 motifs were represented only in normoxia (Fig. 4A, Fig. S2), whereas FOXL1 motifs were only seen under hypoxic GR binding events (Fig. S3). We found no effect of hypoxia on transcript abundance of SP1, NFIC or FOXL1; however, there was a modest reduction in KLF4 transcript in hypoxia (Fig. 4C). Further analysis revealed loss of KLF4, but not SP1 proteins under hypoxia (Fig. 4D), identifying hypoxia regulation of KLF4.

### 3.5 MicroRNAs regulate the impaired GR transactivation in hypoxia

**Fig. 5:**
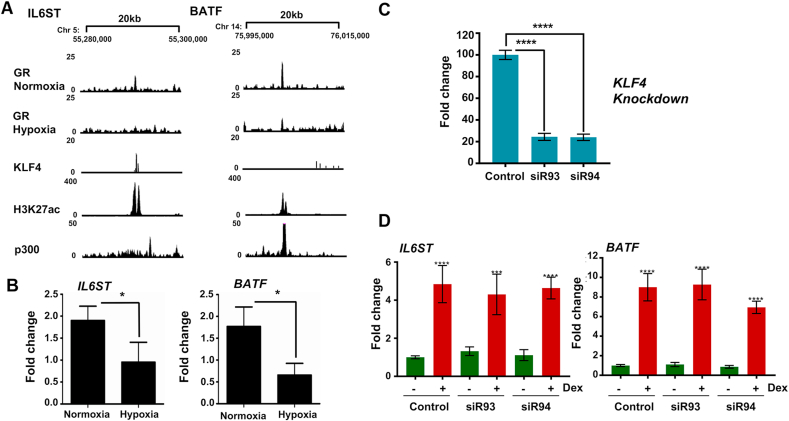
**MicroRNAs regulate the impaired GR transactivation in hypoxia**. (A) Cistrome for KLF4 was also aligned with GR peaks, highlighted IL6ST and BATF as genes under common regulation. Individual UCSC browser gene tracks for IL6ST and BATF are shown with GR, KLF4, H3K27ac and p300 peaks. (B) Cells were cultured in normoxia or hypoxia overnight, then treated with control or 100 nM dexamethasone for 4 h before harvest. Expression levels of either IL6ST or BATF transcript were measured by qRT-PCR. Graphs show the fold change of transcripts in response to treatment with dexamethasone compared to control. Mean ± S.D. n = 3. (C) HeLa cells were transfected with KLF4-specific siRNAs (siR93 and 94) and a negative control siRNA for 72 h. KLF4 knockdown efficiency was measured by qRT-PCR. Representative data are shown as mean ± S.E.M. n = 8. (D) Following KLF4 knockdown, cells were treated with dexamethasone (Dex) or DMSO as the vehicle. ChIP-qPCR was performed using IL6ST and BATF specific primers. CT values were normalised to Spike-In chromatin. Data are presented as fold enrichment over the Veh-treated control group. Data are shown as mean ± S.E.M. n = 4. ****p ≤ 0.0001, ***p ≤ 0.001, *p < 0.05.

To further study the role of KLF4 in the GR cistrome, UCSC browser gene tracks for candidate genes were examined according to the defined histone H3K27ac and co-activator p300 ChIP-seq data, as well as previously published KLF4 ChIP-seq data (GSM447584 (Lister et al., 2009)). Interestingly, both overlapping GR/KLF4 binding sites (IL6ST) and adjacent GR/KLF4 binding sites (BATF) (Fig. 5A) were observed. Consistent with this, qRT-PCR showed a reduction of Gc induced GR transactivation of both IL6ST and BATF in hypoxia (Fig. 5B).

**Fig. 6:**
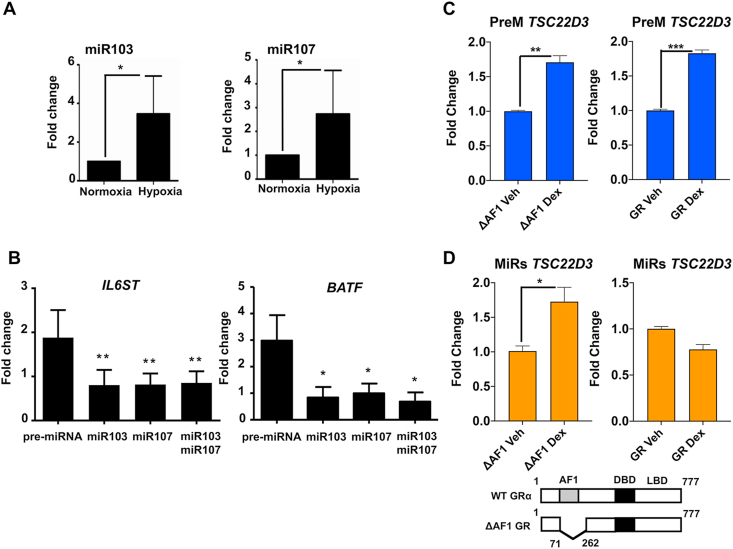
**MicroRNAs impact on GR transactivation function**. (A) Cells were cultured in normoxia or hypoxia overnight, and the expression of both microRNAs 103 and 107 (miR103 and miR107) measured by qRT-PCR. Mean ± S.D. n = 3, *p < 0.05. (B) Cells were transfected with pre-microRNA control, or 50 nM mimics for either hsa-miR-103a-3p or hsa-miR-107, or both together, for 48 h. Cells were then treated with 100 nM dexamethasone or DMSO for 4 h prior to RNA purification. Expression levels of either IL6ST, or BATF transcript were measured by qRT-PCR. Data are shown as fold change of transcripts in response to dexamethasone treatment over DMSO control. Mean ± S.D. n = 3. **p < 0.001, *p < 0.05. (C, D) HEK293T cells were transfected with either control sequences (PreM), or microRNA mimic (MiRs) for 48 h. Following transfection with ΔAF1 (GR construct lacking N-terminus), or full-length GR overnight, cells were treated with vehicle or 100 nM dexamethasone (Dex) for 4 h before harvest. RNA samples were purified and analyzed by qRT-PCR for the TSC22D3 mRNA. Data are shown as mean ± S.E.M. n = 3. ***p < 0.001, **p < 0.01, *p < 0.05.

To determine if hypoxia impaired GR transactivation is due to loss of KLF4 expression, an independent KLF4 knockdown study was performed (Fig. 5C). Subsequently, ChIP-qPCR was completed using IL6ST and BATF primers targeting the GR binding sites (Fig. 5D). Unexpectedly, dexamethasone-induced GR binding activity to the IL6ST and BATF sites was unchanged after depletion of KLF4 (Fig. 5D). KLF4 is known to be a target for microRNAs 103 and 107 (Chen et al., 2012) and both of these microRNAs are induced by hypoxia in a variety of cell types (Kulshreshtha et al., 2007). Therefore, the expressions of both microRNA103 and 107 were measured, and indeed, in this cell system, both microRNAs were significantly induced by hypoxic culture (Fig. 6A). Furthermore, overexpression of microRNA103 and 107 mimetics in normoxia impaired GR transactivation of both IL6ST and BATF (Fig. 6B), recapitulating the effect of hypoxia culture. These results suggest that KLF4 alone is not sufficient to explain the hypoxia regulation of GR chromatin engagement. The microRNAs 103 and 107 are closely related, and share a major overlap in target gene preference.

### 3.6 MicroRNA 103 and 107 regulation of GR transactivation function

**Fig. S4:**
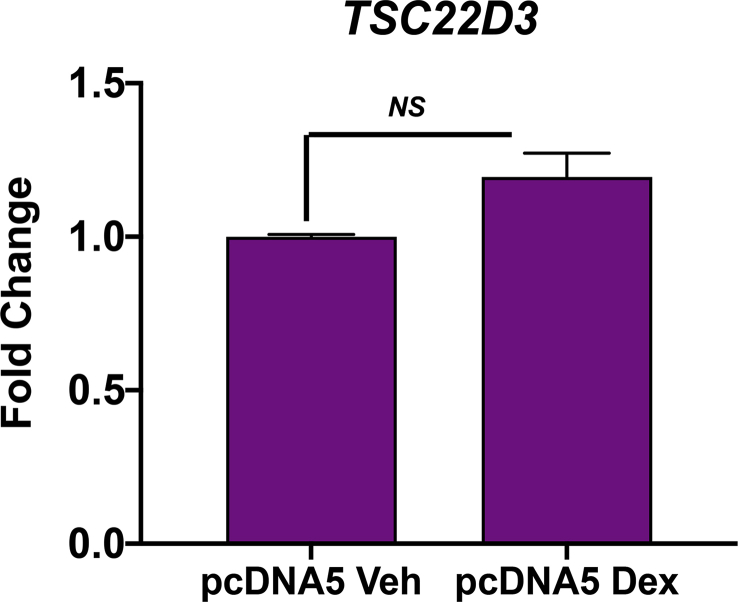
**The expression of TSC22D3 transcript is not affected by our transfection method.** HEK293T cells were transfected with control sequences (PreM) for 48 h, together with transfection using pcDNA5 construct overnight. Following, cells were treated with either vehicle or 100 nM dexamethasone (Dex) for 4 h before harvest. RNA was purified and analyzed by qRT-PCR. Data are shown as mean ± S.E.M., n = 3. *NS*: not significant.

**Fig. S5:**
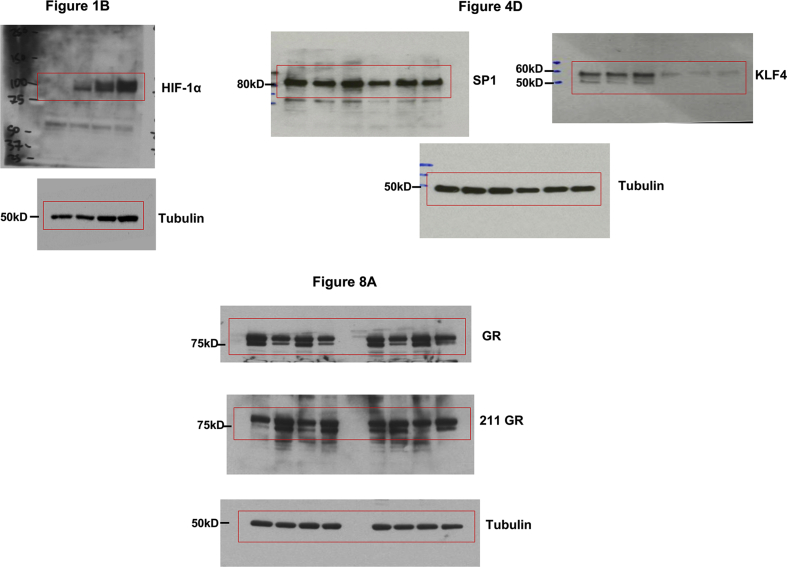
**Original scans of the** immunoblots **shown in paper (boxed bands).** Data are corresponding to Fig. 1, Fig. 4, Fig. 8A.

The identification of the two microRNAs as novel regulators of GR transactivation prompted us to investigate transactivation domain function. We moved to HEK293T cells, which are insufficient in endogenous GR expression to drive endogenous target gene induction (Fig. S4). We analyzed the expression of the index target gene TSC22D3 (or GILZ), identified as a transactivated target gene sensitive to hypoxia, cluster 1 in Fig. 1D. Importantly, GR induction of the TSC22D3 gene requires only the AF2 domain of the GR, permitting analysis of the role of GR AF1 in mediating inhibition by the microRNAs (Fig. 6C) (Hong et al., 1997; Teyssier et al., 2006). However, although the microRNA 103/107 opposed full-length GR transactivation of TSC22D3, they lacked the same effect when transactivation was driven by a GR lacking the AF1 domain, suggesting that the inhibitory effect required the presence of the GR N terminal transactivation domain (Fig. 6D).

**Fig. 7:**
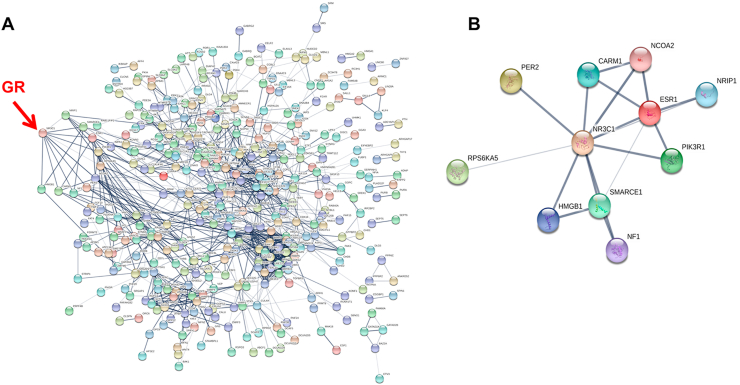
**MicroRNAs regulate a functional network of genes related to the glucocorticoid receptor**. (A) The full list of microRNA103 and 107 target genes was analyzed by STRING10 in order to identify connections with the glucocorticoid receptor. A functional and highly interconnected network was identified, which is depicted. The full list of target genes used for the analysis is in Table S5. (B) The closest, functional connections between the microRNA 103/107 targeted gene network, and the glucocorticoid receptor (NR3C1) identified by STRING10 analysis are depicted. These include the GR co-modulators CARM1 and NCOA2.

These two microRNAs target many genes in a functional, coherent network related to the glucocorticoid receptor (Fig. 7A and B, Tables S4 and S5), including the GR co-modulators CARM1, and NCOA2. As can be seen from the predicted genetic targets of the two microRNAs KLF4 is a direct target, which is supported by earlier work showing a loss of KLF4 protein in cells expressing the microRNA 103/107 (Chen et al., 2012). Therefore, it appears that hypoxic stabilization of HIF-1α drives expression of the microRNA 103/107, and that this intermediary step leads to loss of KLF4 protein expression. However, the KLF4 protein loss is associated with the loss of GR function, rather than being the sole cause of it.

### 3.7 Hypoxia regulates GR function through transactivation domain

**Fig. 8:**
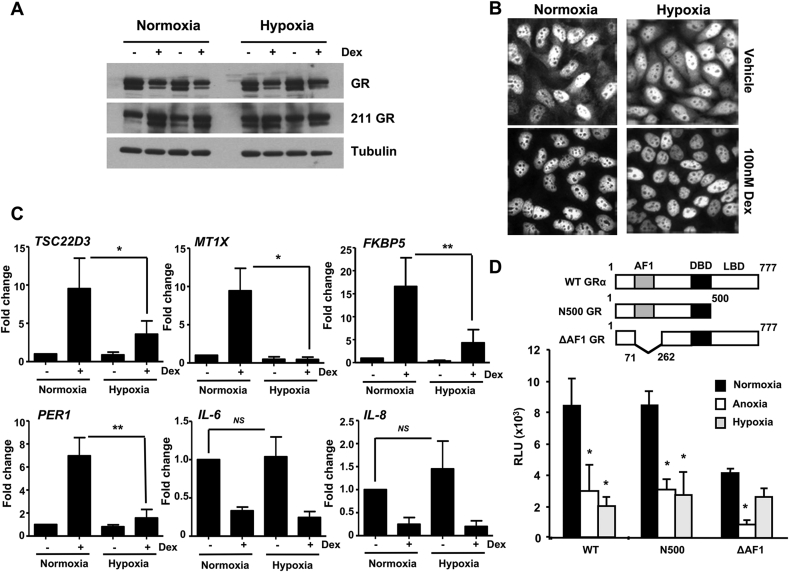
**Hypoxia regulates GR function through transactivation domain**. (A) HeLa cells were cultured in either normoxia or hypoxia overnight, treated with 100 nM dexamethasone (Dex) for 1 h then lysed and immunoblotted for GR and Ser211 phosphorylated GR. Tubulin was used as a loading control. Samples from two independent experiments are shown. (B) Cells cultured in normoxia or hypoxia overnight were treated with 100 nM dexamethasone (Dex) then fixed, and immunolabelled for GR (white) expression. (C) Cells were cultured under normoxic or hypoxic conditions overnight, then treated with dexamethasone for 4 h prior to lysis. qRT-PCR was used to quantify GR transactivation of TSC22D3, MT1X, FKBP5, and PER1 and transrepression of IL-6 and IL-8. Graphs show mean ± S.D. of experiments performed in triplicate and repeated three times. **p < 0.001, *p < 0.05, compared to normoxia. *NS*: not significant. (D) HEK293T cells expressing 2 μg TAT3-luc together with full length wild type receptor, GR ΔAF1 (lacking N-terminus), or GR N500 (lacking C-terminus) were cultured under normoxia with vehicle or 100 μM deferoxamine (hypoxia), or cultured in anoxia overnight. Cells were incubated with dexamethasone for 16 h before luciferase assay. Graph depicts mean ± S.D. and is representative of at least three independent experiments. *p < 0.05 compared to normoxia.

The earliest response of the GR to ligand activation is phosphorylation, a step required for full activity of the activation domains on some enhancers and nuclear translocation to permit engagement with target sites in the genome. These two steps were investigated under hypoxic conditions. Neither Ser211 phosphorylation (Fig. 8A), nor nuclear accumulation (Fig. 8B) was affected by hypoxia, implying a post-nuclear event; in keeping with the differential engagement with target sites, rather than loss of GR binding to DNA.

As the two transactivation domains of the GR may be differentially impacted by the microRNA 103/107. Therefore, we wanted to determine if there were domain-dependencies in the hypoxia response. Initially examined four GR transactivated genes, which have been shown a differential recruitment of GR in hypoxia (Fig. 8C). Interestingly, we saw a loss of GR transactivation in all four genes, despite the previously observed variation in GR recruitment, and GR site H3K27 acetylation. This result further suggests that the GR transactivation domains may be affected by oxygen tension, as well as the selection of binding sites. This diversity of mechanisms may explain the previous, contradictory findings in the literature. In addition, we selected two index GR transrepressed genes, inflammatory cytokines IL-6 and IL-8 (Fig. 8C). The lack of impact of hypoxia on GR transrepression of IL-6 and IL-8 further supports a role for hypoxia in regulating GR transactivation, independently of GR transrepression.

Because we had evidence that the GR AF1 domain was the target for the microRNAs (Fig. 6D), we further tested the function of the two GR transactivation domains separately using a simple reporter gene capable to responding to either domain in isolation (Fig. 8D). Loss of the ligand-binding domain resulted in a constitutive transactivating molecule sensitive to both anoxia and, the hypoxia mimetic deferoxamine. Strikingly, loss of the N terminal AF1 transactivation domain attenuated the inhibitory effect of the hypoxia mimetic, although anoxia still had a profound inhibitory effect. These results highlight an additional mechanism whereby hypoxia regulates GR transactivation through AF1 domain. As this is the same domain affected by the microRNAs 103 and 107 (Fig. 6D), it provides evidence that the hypoxia induction of the microRNAs is a plausible mechanism resulting in an AF1-specific defect in GR transactivation.

## 4 Discussion

Gcs exert a broad spectrum of metabolic and immune regulatory effects through activation of the near-ubiquitously expressed GR (Yang et al., 2012). The mechanisms responsible for conferring cell-type specificity of action are emerging, with up to 90% of GR binding sites being cell specific (Wiench et al., 2011a). This degree of specificity can be directed by epigenetic regulation of changes in accessibility of cis-elements (Wiench et al., 2011b). The cellular environment is also important for regulating responses, e.g. at sites of inflammation. This may be explained in part by recent observations that proinflammatory signalling regulates the GR cistrome, opening new sites and closing off others (Uhlenhaut et al., 2013). Sites of inflammation differ in several respects from healthy tissue, but amongst the changes consistently observed is the reduction in oxygen tension, due to bacterial and immune cell oxygen consumption (Oliver et al., 2009). Indeed, macrophages are specifically adapted to migrate towards hypoxia, and utilise primarily glycolytic ATP generation which is not dependent on abundant oxygen availability (Garedew et al., 2010; Ruiz-Garcia 2011). Previous reports yielded conflicting results on the effects of hypoxia on Gc action, possibly due to methodological differences, and reliance on a limited number of end-points.

Our initial studies showed a marked reduction in GR transactivation of a simple reporter gene with consensus GRE elements. Our failure to detect physical interaction between the major hypoxia responsive transcription factor, HIF-1α, suggested that the mechanisms of hypoxia adaptation are more complex and diverse, and so we undertook a genome-wide analysis of GR binding. We found most GR binding events were not in the proximal promoter regions of coding sequences, as reported before, and that peaks of GR-binding sites were evenly distributed between upstream and downstream sequences (So et al., 2007). Remarkably, hypoxia opened a new GR cistrome, with a number of GR binding sites shared in common with normoxic conditions. Nearly 50% of GR binding sites were lost in the transition to hypoxia, indicating the re-programming of the GR cistrome in response to changes in ambient oxygen tension. This specificity of effect, which may be further influenced by cell-type specific regulatory mechanisms, potentially explains the discrepant findings reported to date in the literature (Charron et al., 2009; Kodama et al., 2003; Leonard et al., 2005; Sengupta and Wasylyk, 2004; Wang et al., 2012).

For the first time, we identified GR cistrome-regulated genes in both normoxia and hypoxia. Using GREAT analysis, we found normoxic GR binding sites were strongly associated with genes encoding components of the inflammatory response and apoptosis. Strikingly, hypoxia rewires the GR cistrome. Gene ontology analysis identified the terms of cellular response to oxygen tension and plasma membrane long-chain fatty acid transport, supporting a reprogramming of Gc action dependent on oxygen tension (McLean et al., 2010). Interestingly, further disease ontology analysis revealed hypoxic GR-cistrome is associated with control of arthritis. As inflammatory arthritis is itself associated with reduced oxygen tension, this may point to an evolutionary adaptation to Gc signalling under hypoxic conditions to promote resolution of inflammation.

Further analysis of GR binding sites by ChIP-PCR confirmed the predicted changes in GR recruitment, with coordinate changes in histone H3K27ac status, suggesting productive engagement of target chromatin by the GR. Interestingly, target gene transactivation was consistently inhibited under hypoxic conditions, a change that was not predicted based on GR recruitment. This suggests the involvement of additional mechanisms explaining differential gene expression, possibly resulting from differential engagement of co-modulators, or additional effects conferred by other uncharacterised cis-elements.

As predicted, motif discovery analysis identified GRE motifs under both normoxia and hypoxia. Surprisingly, although HIF-1α protein can bind in proximity to the GR under hypoxic conditions (Elsby et al., 2009), HIF-1α motif was not shown among the most centrally enriched motifs associated with hypoxic GR-cistrome. Instead, hypoxia resulted in less Kruppel-like factor 4 (KLF4) consensus sequences associated with GR binding sites, and increased abundance of FOXL1 consensus sites. This led us to focus on these two transcription factors as potentially involved in modulating GR function in hypoxia. The KLF4 cistrome showed some overlap with the GR cistrome, with both adjacent and overlapping target genomic sequences being identified, potentially indicating a role for KLF4 as a pioneer, or co-binding factor. Previous studies have identified a critical role for AP1 transcription factor in opening genomic binding sites to the GR (Biddie et al., 2011). However, in our analysis we did not detect a change in AP1 site enrichment dependent on oxygen tension.

KLF4, a zinc finger transcription factor, is expressed in epithelia (Shields et al., 1996). It has been defined to play an important role to regulate cell proliferation, migration and differentiation (Ghaleb et al., 2011). Interaction between GR and KLF4 has been observed *in vivo*, where corticosteroid treatment, and expression of KLF4 co-ordinately accelerated skin barrier function acquisition, and it was shown that genes regulated by GR and KLF4 significantly overlap (Patel et al., 2006). Studies in colorectal carcinoma metastases revealed loss of KLF4 protein expression driven by induction of two microRNAs 103 and 107 (Chen et al., 2012). In our own studies presented here, we also observed a loss of KLF4 expression in hypoxia, which was associated with partial loss of the normoxic GR cistrome. Although KLF4 knockdown had no impact on GR recruitment to specific GREs, both of the microRNAs replicated the loss of GR transactivation seen in hypoxia. Probably, depletion of KLF4 alone is insufficient to replicate the hypoxia effect limiting GR recruitment to its recognition sites, and therefore additional mechanisms downstream of the microRNAs are likely to contribute.

Indeed, the two closely related microRNAs target a broad network of genes with functional connections to GR. Amongst those with the closest links are the co-modulators CARM1, and NCOA2 (also known as SRC2, or GRIP1). Additional evidence for a GR transactivation domain mechanism comes from the GR domain deletion studies on both endogenous, and synthetic reporter genes. Here we see a requirement for the AF1 domain to transmit the hypoxia/microRNA 103/107 signal. Now, the two GR transactivation domains are known to functionally interact, with some co-modulators such as NCOA2 binding to both, and inducing structural modification to the intrinsically-disordered AF1 domain (Weikum et al., 2017). Therefore, we propose that loss of co-modulators resulting from microRNA action, leads to a defective AF1 conformation which through allosteric interaction impairs the transactivation function of AF2. Deletion of the AF1, as in the ΔAF1 mutant, frees the AF2 from this negative effect, and permits transactivation even in hypoxia, likely through a spectrum of co-modulators that are not affected by the hypoxia-microRNA circuit.

**Fig. 9:**
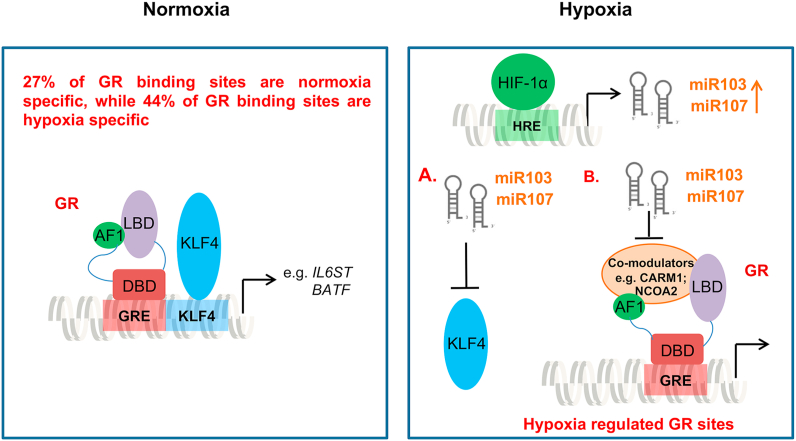
**A graphical overview of GR function under normoxia or hypoxia, and the role ofmicroRNA103/107 and KLF4**. Left: GR transactivation of targets such as IL6ST, and BATF under normoxia, where the KLF4 binding motif was identified by our ChIP-seq analysis. Right: Hypoxia drives expression of both microRNAs 103 and 107 (miR103 and miR107), which represses KLF4 expression (as shown in panel A), and inhibits multiple GR co-modulators, including CARM1 and NCOA2, as shown in panel B.

Therefore, we propose that the effector molecules for the change in Gc sensitivity, and target gene specificity, in hypoxia are these two key microRNAs. Moreover, our data suggest a complex system-wide effect of the microRNAs altering the expression of other transcription factors, including KLF4, as well as co-modulator genes, including CARM1, and NCOA2. Taken together, our data identified a reprogrammed GR cistrome induced by hypoxia (Fig. 9). In addition to the important role of cell-lineage specifying transcription factors, environmental sensing pathways such as those activated in hypoxia through the regulation of microRNAs are also involved in directing the GR cistrome, and subsequently in regulating GR function. This discovery opens up a novel mechanism to explain the clinically observed variation in Gc response in inflammation.

## Funding

This work was funded in part by the 10.13039/501100000319ARUK. Laura Matthews and Karen Forbes were funded by 10.13039/501100000770University of Manchester Stepping Stones fellowships. David Ray is funded by 10.13039/501100013373NIHR Oxford Biomedical Research Centre, and 10.13039/501100000265MRC programme grant MR/P023576/1. David Ray is a Wellcome Investigator, Wellcome Trust, United Kingdom (107849/A/15/Z).

## CRediT authorship contribution statement

**Nan Yang:** Investigation, Formal analysis, Writing - review & editing. **Andrew Berry:** Investigation. **Carolin Sauer:** Investigation. **Matthew Baxter:** Formal analysis. **Ian J. Donaldson:** Formal analysis. **Karen Forbes:** Resources. **Rachelle Donn:** Conceptualization. **Laura Matthews:** Writing - original draft, Writing - review & editing. **David Ray:** Writing - original draft, Writing - review & editing, Supervision.

## Declaration of competing interest

The authors declare no conflict of interest.

## Figures and Tables

**Table S1:**
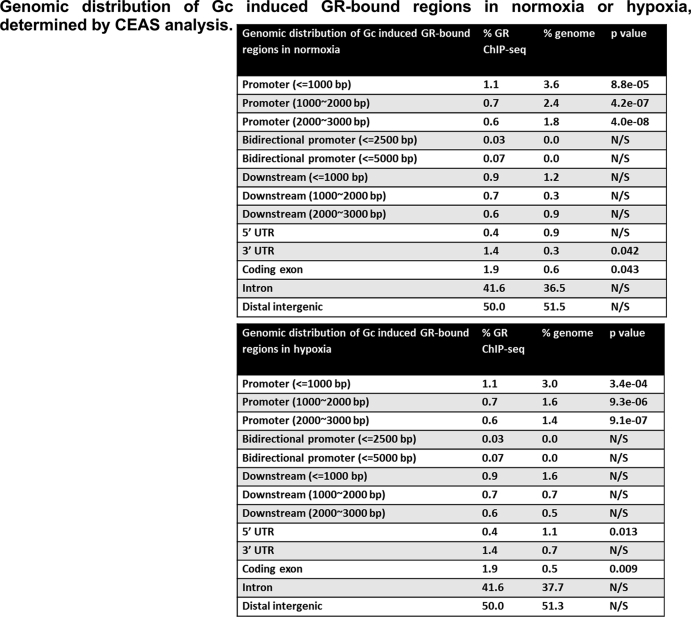
Genomic distribution of Gc induced GR-bound regions in normoxia or hypoxia, determined by CEAS analysis.

**Table S2:**
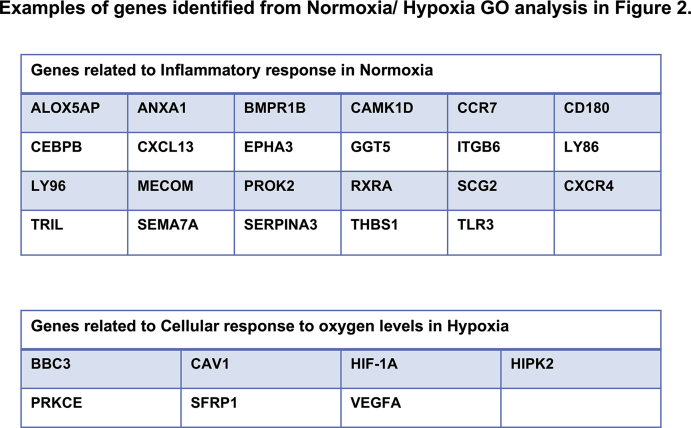
Examples of genes identified from Normoxia/ Hypoxia GO analysis in Fig. 2.

**Table S3:**
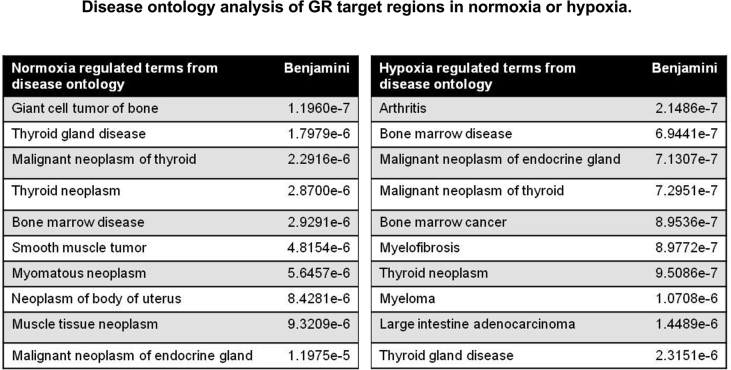
**Disease ontology analysis of GR target regions in normoxia or hypoxia**. Disease ontology analysis was completed using GREAT, version 2.0.2. Top 10 enriched terms from the disease ontology are presented. Benjamini-Hochberg adjusted p values are shown.

**Table S4:**
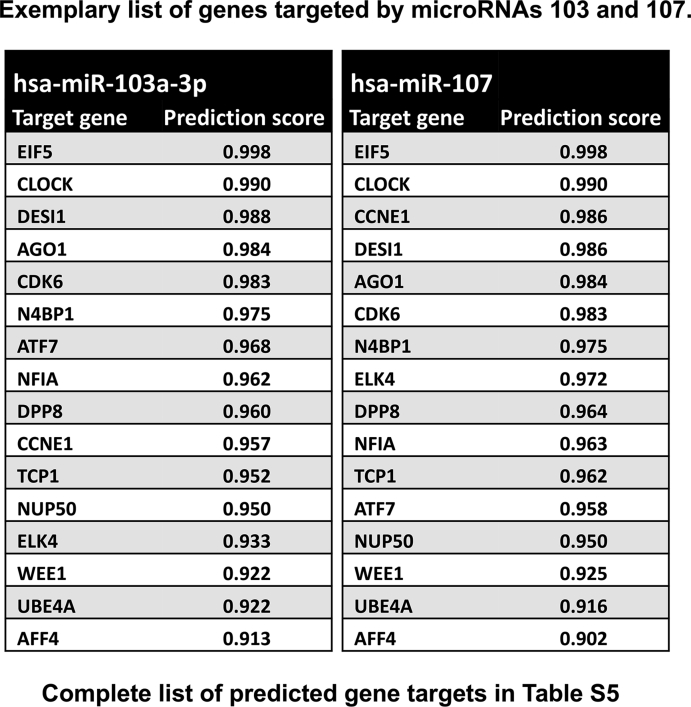
Exemplary list of genes targeted by microRNAs 103 and 107.

## Appendix A Supplementary data

Table S5 Full list of predicted targets for microRNAs 103 and 107. Table S5
